# Sirtuin 3 is essential for hypertension‐induced cardiac fibrosis via mediating pericyte transition

**DOI:** 10.1111/jcmm.15437

**Published:** 2020-05-28

**Authors:** Han Su, Heng Zeng, Bo Liu, Jian‐Xiong Chen

**Affiliations:** ^1^ Department of Pharmacology and Toxicology University of Mississippi Medical Center Jackson MS USA; ^2^ Department of General Surgery Third Xiangya Hospital Central South University Changsha China

**Keywords:** cardiac dysfunction, fibrosis, hypertension, pericytes, reactive oxygen species, sirtuin 3, transforming growth factor beta 1

## Abstract

Hypertension is the key factor for the development of cardiac fibrosis and diastolic dysfunction. Our previous study showed that knockout of sirtuin 3 (SIRT3) resulted in diastolic dysfunction in mice. In the present study, we explored the role of SIRT3 in angiotensin II (Ang‐II)–induced cardiac fibrosis and pericyte‐myofibroblast transition. NG2 tracing reporter NG2‐DsRed mouse was crossed with wild‐type (WT) mice and SIRT3KO mice. Cardiac function, cardiac fibrosis and reactive oxygen species (ROS) were measured. Mice infused with Ang‐II for 28 days showed a significant reduction of SIRT3 expression in the mouse hearts. Knockout of SIRT3 sensitized Ang‐II‐induced elevation of isovolumic relaxation time (IVRT) and reduction of ejection fraction (EF) and fractional shortening (FS). Ang‐II‐induced cardiac fibrosis, capillary rarefaction and hypertrophy were further enhanced by knockout of SIRT3. NG2 pericyte tracing reporter mice infused with Ang‐II had a significantly increased number of NG2‐DsRed pericyte in the heart. Knockout of SIRT3 further enhanced Ang‐II‐induced increase of pericytes. To examine pericyte‐myofibroblast/fibroblast transition, DsRed pericytes were co‐stained with FSP‐1 and α‐SMA. Ang‐II infusion led to a significant increase in numbers of DsRed^+^/FSP‐1^+^ and DsRed^+^/α‐SMA^+^ cells, while SIRT3KO further developed pericyte‐myofibroblast/fibroblast transition. In addition, knockout of SIRT3 promoted Ang‐II‐induced NADPH oxidase‐derived ROS formation together with increased expression of transforming growth factor beta 1 (TGF‐β1). We concluded that Ang‐II induced cardiac fibrosis partly by the mechanisms involving SIRT3‐mediated pericyte‐myofibroblast/fibroblast transition and ROS‐TGF‐β1 pathway.

## 1 INTRODUCTION

Hypertension is a progressive cardiovascular disease with high prevalence of heart failure. Cardiac pressure overload induced by hypertension causes cardiac remodelling such as capillary rarefaction, cardiac fibrosis and hypertrophy.^1^, ^2^, ^3^, ^4^ Among these changes, fibrosis including perivascular and myocardial fibrosis contributes essentially to diastolic dysfunction, which is one of the leading causes of heart failure with preserved ejection fraction (HFpEF).^1^, ^5^, ^6^ So far, the mechanisms of how hypertension causes cardiac fibrosis remain incompletely understood.

Sirtuin 3 (SIRT3) is a key regulator for deacetylation of mitochondrial proteins. SIRT3 has been shown to regulate a variety of physiological and pathological processes including metabolic homeostasis, oxidative stress, apoptosis and ageing.^7^, ^8^, ^9^, ^10^, ^11^ Study has shown that SIRT3 prevents tubule‐interstitial fibrosis in hypertensive kidney.^12^ Our previous studies have revealed that endothelial‐specific SIRT3 deletion triggers cardiac remodelling and impairs diastolic dysfunction in mice via reprogramming endothelial metabolism in which increased oxidative stress plays an important role.^1^, ^5^ Pericyte was identified as the progenitor of myofibroblast and fibroblast that contributes to the deposition of extracellular matrix (ECM).^13^, ^14^, ^15^, ^16^, ^17^, ^18^, ^19^ Studies have shown that pericyte‐myofibroblast/fibroblast transition is a novel mechanism contributing to the fibrosis in the kidney. Pericyte‐myofibroblast/fibroblast transition is essentially in the pathological process of tumour invasion and metastasis.^15^, ^20^, ^21^ Although both SIRT3 and pericytes play a crucial role in fibrosis, the association between these two in hypertension‐induced cardiac fibrosis has not been defined.

In the present study, we aim to investigate the functional roles of SIRT3 in cardiac fibrosis in response to hypertension. We found that Ang‐II infusion reduced SIRT3 expression in the mouse heart. Knockout of SIRT3 further sensitized Ang‐II‐induced cardiac fibrosis, capillary rarefaction, hypertrophy and cardiac dysfunction. Pathology of fibrosis in hypertensive heart was strongly associated with pericyte transition into fibrotic cells. In addition, Ang‐II‐induced TGF‐β1 expression and ROS formation were further enhanced by knockout of SIRT3.

## 2 MATERIALS AND METHODS

All procedures conformed to the Institute for Laboratory Animal Research Guide for the Care and Use of Laboratory Animals. It was also approved by the Animal Care and Use Committee of University of Mississippi Medical Center (Protocol ID: 1280B). The investigation conformed to the National Institutes of Health (NIH) Guide for the Care and Use of Laboratory Animals (NIH Pub. No. 85‐23, Revised 1996).

### 2.1 Experimental animal model and treatment

Wild‐type (WT) control, SIRT3 knockout (SIRT3KO) (Stock#012755) mice and NG2 tracing reporter NG2‐DsRedBAC (Cspg4‐DsRed) (Stock#008241) mice were obtained from the Jackson Laboratory (Jackson Laboratory) and were bred by our laboratory. SIRT3KO mice were determined by polymerase chain reaction using the following primers: SIRT3KO common, 5′‐CTT CTG CGG CTC TAT ACA CAG‐3′; SIRT3KO wild‐type reverse, 5′‐TGC AAC AAG GCT TTA TCT TCC‐3′; SIRT3KO mutant reverse, 5′‐TAC TGA ATA TCA GTG GGA ACG‐3′. NG2‐DsRedBAC mice were determined using primers: NG2‐DsRed transgene forward, 5′‐TTC CTT CGC CTT ACA AGT CC‐3′; NG2‐DsRed transgene reverse, 5′‐GAG CCG TAC TGG AAC TGG‐3′; NG2‐DsRed positive control forward, 5′‐CTA GGC CAC AGA ATT GAA AGA TCT‐3′; NG2‐DsRed positive control reverse, 5′‐GTA GGT GGA AAT TCT AGC ATC ATC C‐3′ (Integrated DNA Technologies). SIRT3KO mice were crossed with NG2‐DsRedBAC mice. Among these crossed mice, homozygous SIRT3KO mice were selected by PCR firstly. Then, NG2‐DsRed‐SIRT3KO mice were selected from homozygous SIRT3KO mice (Figure S1). Experiments were performed on male mice at 4‐7 months of age.

To induce hypertension, both WT mice and SIRT3KO mice were infused with Ang‐II (1000 ng/kg/min) for 28 days via subcutaneously implanted Alzet miniosmotic pumps (DURECT Corporation) while under anaesthesia.^22^, ^23^

### 2.2 Measurement of blood pressure

Systolic blood pressure, diastolic blood pressure and mean arterial pressure (MAP) were measured by tail‐cuff occlusion method according to the manufacturer's instructions (Kent Scientific Corporation). Before and after implantation of Ang‐II pumps, measurements were taken once a day at the approximate same time each day. We took the first 3 days as an adjustment time for mice, and the results were not included. Blood pressure measurements for remaining days were averaged for the final results.^24^

### 2.3 Echocardiography

We performed transthoracic echocardiograms on WT mice, SIRT3KO mice, WT mice + Ang‐II and SIRT3KO mice + Ang‐II at day 28 after Ang‐II infusion using the Vevo 3100 Imaging System (VisualSonics Inc). CFR, EF, FS and IVRT were measured at the approximate same time.^25^

### 2.4 Histological and immunofluorescence analysis

The heart tissues were fixed in neutral‐buffered 10% formalin solution (SF93‐20; Fisher Scientific) and embedded in frozen OCT compound (4585; Fisher Health Care). Frozen sections and paraffin sections were prepared (10 µm in thickness). All the heart tissues were prepared under the same conditions. Haematoxylin and eosin (H&E) staining and Masson's trichrome staining (paraffin section) were performed to measure cardiomyocyte size and the degree of fibrosis (blue) in the heart. ROS (frozen sections) was measured by DHE staining. Some fresh frozen sections were used to observe DsRed^+^ cells (1:150; Abcam). Also, some frozen sections were immunostained with IB4 for endothelial cells (ECs) (Invitrogen), NG2, PDGFR‐β for pericytes (1:150; Abcam) and α‐SMA, FSP‐1 and collagen‐I for fibrotic cells (1:150; Abcam). Other frozen sections were immunostained with TGF‐β1 primary antibody (1:50; Santa Cruz). These immunostained sections were incubated with second antibodies conjugated with fluorescein isothiocyanate (FITC) or Cy3 (1:500). A Nikon microscope, Nikon digital camera and Nikon software (Nikon) were used to capture images. Six random microscopic fields were analysed using Image Analysis Software (ImageJ, NIH). Calculation of pericytes was presented as NG2‐DsRed^+^, DsRed^+^ and PDGFR‐β^+^ cells numbers/total DAPI^+^ nuclei numbers. Calculation of fibrotic cells was presented as FSP‐1^+^ and α‐SMA^+^ cells numbers/total DAPI^+^ nuclei numbers.

### 2.5 Western blot analysis

Mouse left ventricle heart tissues were collected and homogenized in lysis buffer. The homogenates were centrifuged at 16 000 *g* at 4°C for 15 minutes. The BCA protein assay kit (Pierce Co) was used to analyse the protein concentrations. Equal amounts (20 µg) of the protein were separated by 10% SDS‐PAGE gel and transferred to a polyvinylidene difluoride (PVDF) membrane. The membranes were blocked with 5% non‐fat dry milk in Tris‐buffered saline and incubated with the following primary antibodies overnight: SIRT3 (1:1000; Cell Signaling), β‐MHC (1:1000; Abcam), TGF‐β1 (1:1000), gp91^phox^ and p47^phox^ (1:1000; BD transduction). After washing, the membranes were incubated for 2 hours with an anti‐rabbit or anti‐mouse secondary antibody coupled to horseradish peroxidase (1:5000; Santa Cruz). Densitometric analyses were carried out with image acquisition and analysis software (Bio‐Rad).

### 2.6 Statistical analysis

Data are expressed as mean ± SEM The significance of differences in the means of corresponding values among groups was determined by using the one‐way ANOVA. Significance of differences among groups was determined using multiple comparison. The significance of differences between two groups was determined by Student's *t* test. *P* < .05 was considered to be significant. Data were analysed with Prism software, v.8.0 (GraphPad Software).

## 3 RESULTS

### 3.1 Ang‐II reduced SIRT3 levels in the mouse hearts

**FIGURE 1:**
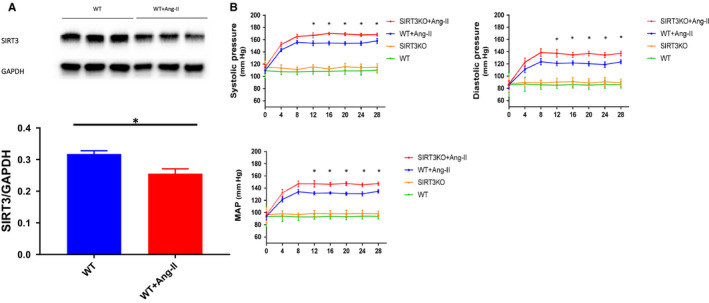
Hypertension interacted with SIRT3 expression. A, SIRT3 expression was decreased in WT mice + Ang‐II (n = 3 mice) compared to WT mice (n = 3 mice). Mean ± SEM, *P < .05. B, Systolic pressure, diastolic pressure and mean arterial pressure (MAP) were significantly enhanced in WT mice + Ang‐II compared to WT mice (n = 9 mice), and knockout of SIRT3 further increased these elevations (n = 9 mice). Mean ± SEM, *P < .05

To explore the interaction of SIRT3 and Ang‐II‐induced cardiac fibrosis, we first examined the role of Ang‐II on SIRT3 expression in the mouse heart. As shown in Figure 1A, Ang‐II infusion resulted in a significant reduction of SIRT3 expression in mouse heart. In addition, WT mouse infusion with Ang‐II significantly increased blood pressure, including systolic pressure, diastolic pressure and mean arterial pressure (MAP). In comparison with WT mice + Ang‐II, the blood pressure was further elevated in SIRT3KO mice + Ang‐II (Figure 1B).

### 3.2 Knockout of SIRT3 sensitized Ang‐II‐induced cardiac dysfunction in mice

**FIGURE 2:**
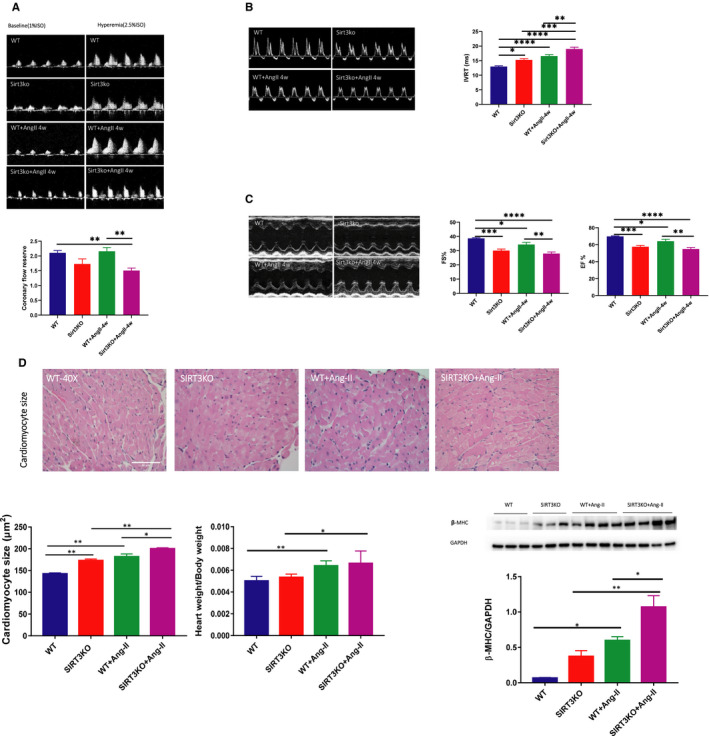
Knockout of SIRT3 enhanced Ang‐II‐induced cardiac hypertrophy and dysfunction. A, No significant difference was found between WT mice and WT mice + Ang‐II. Decreased CFR was found in SIRT3KO mice + Ang‐II compared to WT mice + Ang‐II (n = 11‐12 mice). Mean ± SEM, *P < .05, **P < .01, ****P < .0001. B, Ang‐II treatment impaired diastolic function as evidence by increases in IVRT in comparison with WT mice. Knockout of SIRT3 further enhanced Ang‐II‐induced elevation of IVRT (n = 13‐5 mice). Mean ± SEM,*P < .05, **P < .01, ***P < .001, ****P < .0001. C, Knockout of SIRT3 resulted in a further decline of EF and FS induced by Ang‐II infusion (n = 13‐5 mice). Mean ± SEM *P < .05, ***P < .001, ****P < .0001. D, HW/BW was increased in WT mice + Ang‐II compared to WT mice. Cardiomyocyte size was increased by Ang‐II compared to WT mice. Significant difference was found between WT mice + Ang‐II and SIRT3KO mice + Ang‐II. Ang‐II up‐regulated expression of β‐MHC in SIRT3KO mice + Ang‐II (n = 4 mice) in comparison with SIRT3KO mice (n = 3 mice). Knockout of SIRT3 also increased expression of β‐MHC after Ang‐II infusion (n = 4 mice) compared to WT mice + Ang‐II (n = 4 mice). Mean ± SEM, *P < .05, **P < .01

Measurement of coronary blood flow reserve (CFR) showed that there was no significant difference of CFR between WT mice and WT mice + Ang‐II. Knockout of SIRT3 caused a significant reduction of CFR after Ang‐II infusion (Figure 2A). Infusion with Ang‐II further led to a cardiac dysfunction as evidence by an increase in IVRT and decreases in EF and FS. Knockout of SIRT3 significantly exacerbated Ang‐II‐induced cardiac dysfunction in mice (Figure 2B,C).

### 3.3 Knockout of SIRT3 enhanced Ang‐II‐induced cardiac hypertrophy

Both WT mice and SIRT3 KO mice infused with Ang‐II showed a significant increase in heart weight/bodyweight (HW/BW) ratio compared to their control mice without Ang‐II treatment. Surprisingly, no significant difference of HW/BW was found between the WT mice + Ang‐II and the SIRT3KO mice + Ang‐II (Figure 2D). H&E staining revealed a significant increase in cardiomyocyte size in the WT mice + Ang‐II than that of WT mice. Ang‐II‐induced cardiomyocyte hypertrophy was further enhanced by knockout of SIRT3 (Figure 2D). Consistently, the expression of hypertrophic marker β‐MHC was significantly up‐regulated in the WT mice + Ang‐II compared to the WT mice. The expression of β‐MHC was further increased by knockout of SIRT3 (Figure 2D).

### 3.4 Knockout of SIRT3 enhanced Ang‐II‐induced cardiac fibrosis and microvascular rarefaction

**FIGURE 3:**
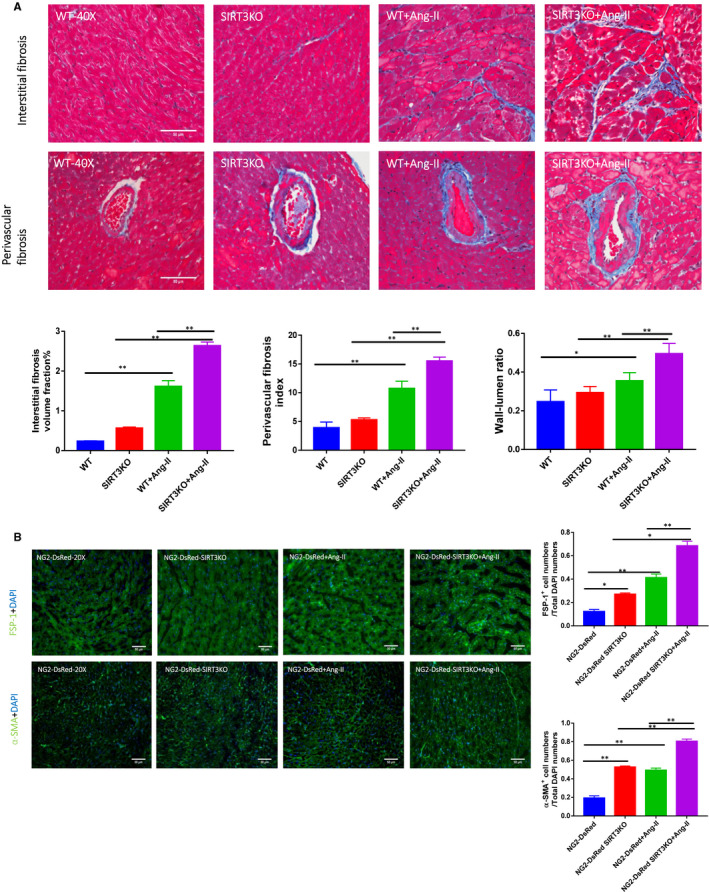
Knockout of SIRT3 accentuated Ang‐II‐induced fibrosis. A, Masson's trichrome staining showed that interstitial fibrosis was increased significantly in WT mice + Ang‐II (n = 4 mice) and knockout of SIRT3 (n = 4 mice) further enhanced this increase as compared to WT mice + Ang‐II. Knockout of SIRT3 promoted Ang‐II‐induced coronary artery remodelling. Perivascular fibrosis and increased wall‐lumen ratio were found in WT mice + Ang‐II (n = 4 mice); these alterations were accentuated by knockout of SIRT3. Mean ± SEM, *P < .05, **P < .01. B, Immunostaining revealed a significant increases in FSP‐1 and α‐SMA levels in NG2‐DsRed mice + Ang‐II (n = 4 mice), and levels of FSP‐1 and α‐SMA were further increased by knockout of SIRT3. Mean ± SEM, *P < .05, **P < .01

The effects of SIRT3 in Ang‐II‐induced cardiac fibrosis were further examined. Masson's trichrome staining showed that there was significantly increased interstitial fibrosis in the WT mice + Ang‐II than that in the WT mice. Knockout of SIRT3 further increased interstitial fibrosis (Figure 3A). Immunostaining study showed a significant enhancement of FSP‐1 and α‐SMA levels in the hearts of NG2‐DsRed mice + Ang‐II compared to that of NG2‐DsRed mice. Ang‐II‐induced fibrosis was further enhanced by knockout of SIRT3 (Figure 3B). Masson's trichrome staining of coronary artery showed increases of wall‐lumen ratio (arterial thickness) and perivascular fibrosis in the WT mice + Ang‐II compared to WT mice. Knockout of SIRT3 further enhanced Ang‐II‐induced wall thickness and perivascular fibrosis (Figure 3A). Moreover, Ang‐II infusion led to a significant decrease in capillary density (IB4 staining), which was further reduced by knockout of SIRT3 (Figure S3). In addition, no IB4^+^/FSP‐1^+^ double‐positive cells were found in the mouse heart (Figure S4).

### 3.5 Knockout of SIRT3 promoted Ang‐II‐induced pericyte recruitment

**FIGURE 4:**
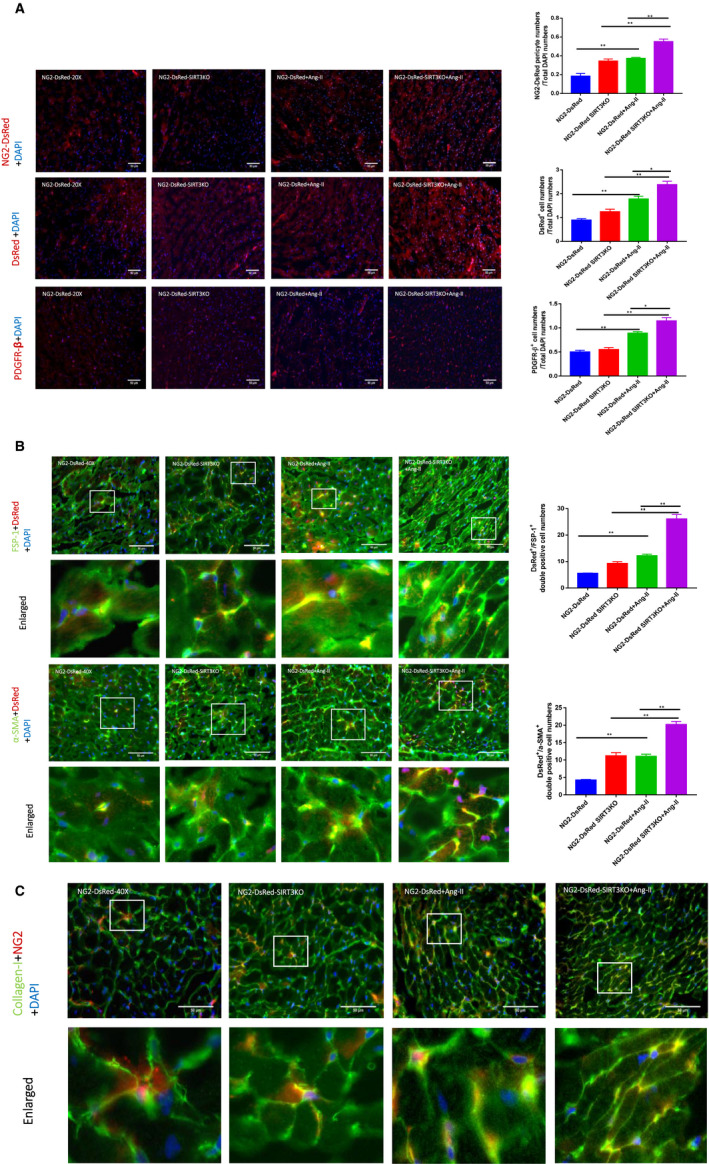
Knockout of SIRT3 promoted Ang‐II‐induced pericyte recruitment and transition. A, Immunostaining data showed there was a significant increase of NG2‐DsRed^+^ pericytes in NG2‐DsRed mice + Ang‐II (n = 4 mice) compared to NG2‐DsRed mice (n = 3 mice). NG2‐DsRed^+^ pericytes were further enhanced in NG2‐DsRed‐SIRT3KO mice + Ang‐II (n = 4 mice) in comparison with NG2‐DsRed mice + Ang‐II (n = 4 mice). Ang‐II significantly enhanced numbers of DsRed^+^ and PDGFR‐β^+^ pericytes; knockout of SIRT3 further enhanced the Ang‐II‐induced changes. Mean ± SEM, *P < .05, **P < .01. B, Knockout of SIRT3 enhanced Ang‐II‐induced pericyte transition. Images and analysis revealing FSP‐1^+^/DsRed^+^ and α‐SMA^+^/DsRed^+^ double‐positive cells in all groups of mice. The numbers of both FSP‐1^+^/DsRed^+^ and α‐SMA^+^/DsRed^+^ double‐positive cells were enhanced in NG2‐DsRed mice + Ang‐II (n = 4 mice) compared to NG2‐DsRed mice (n = 3 mice), and knockout of SIRT3 further developed these increases. Mean ± SEM, *P < .05, **P < .01. C, Images and analysis revealing collagen‐I^+^/NG2^+^ pericytes presented in all groups of mice. The numbers of collagen‐I^+^/NG2^+^ double‐positive cells were enhanced in NG2‐DsRed mice + Ang‐II (n = 4 mice) compared to NG2‐DsRed mice (n = 3 mice), and knockout of SIRT3 further increased collagen‐I^+^/NG2^+^ cells

Using NG2 tracing reporter NG2‐DsRedBAC mouse, we first traced whether Ang‐II infusion increased number of NG2‐DsRed pericyte in mouse hearts. Immunostaining with DsRed and PDGFR‐β was further utilized to validate pericytes. As shown in Figure 4A, both NG2‐DsRed^+^ (traced pericyte) and DsRed^+^ (immune‐stained pericyte) were presented in the mouse hearts. The numbers of NG2‐DsRed^+^ (traced) and DsRed^+^ (immune‐stained) pericyte were significantly increased in the NG2‐DsRed mice + Ang‐II in comparison with NG2‐DsRed mice alone. Knockout of SIRT3 further promoted Ang‐II‐induced pericyte recruitment (Figure 4A). Similarly, the numbers of PDGFR‐β^+^ pericyte were increased in the NG2‐DsRed mice + Ang‐II than that in the NG2‐DsRed mice. Knockout of SIRT3 further increased numbers of PDGFR‐β^+^ pericyte in mouse hearts (Figure 4A).

### 3.6 Knockout of SIRT3 enhanced Ang‐II‐induced pericyte‐myofibroblast transition

Using NG2 tracing reporter NG2‐DsRedBAC mouse, we further traced whether NG2‐DsRed pericyte differentiated into myofibroblasts or fibroblasts in the heart by co‐immune‐stained with fibroblastic markers (FSP‐1 and α‐SMA) and DsRed. FSP‐1^+^/DsRed^+^ and α‐SMA^+^/DsRed^+^ pericytes were presented in the heart of all groups of mice, which indicate the existence of pericyte‐myofibroblast/fibroblast transition (Figure 4B). Furthermore, there were increased numbers of FSP‐1^+^/DsRed^+^, collagen‐I^+^/NG2^+^ and α‐SMA^+^/DsRed^+^ cells in the heart of NG2‐DsRed mice + Ang‐II compared to the NG2‐DsRed mice. Knockout of SIRT3 further enhanced pericyte‐myofibroblasts/fibroblast transition in the hearts (Figure 4B,C). In NG2 tracing reporter NG2‐DsRedBAC mouse, NG2‐DsRed^+^ cells were also presented around the coronary arteries (Figure S2A). In addition, FSP‐1^+^/DsRed^+^ area was increased by Ang‐II infusion and knockout of SIRT3 (Figure S2B).

### 3.7 Knockout of SIRT3 enhanced Ang‐II‐induced TGF‐β1 expression

**FIGURE 5:**
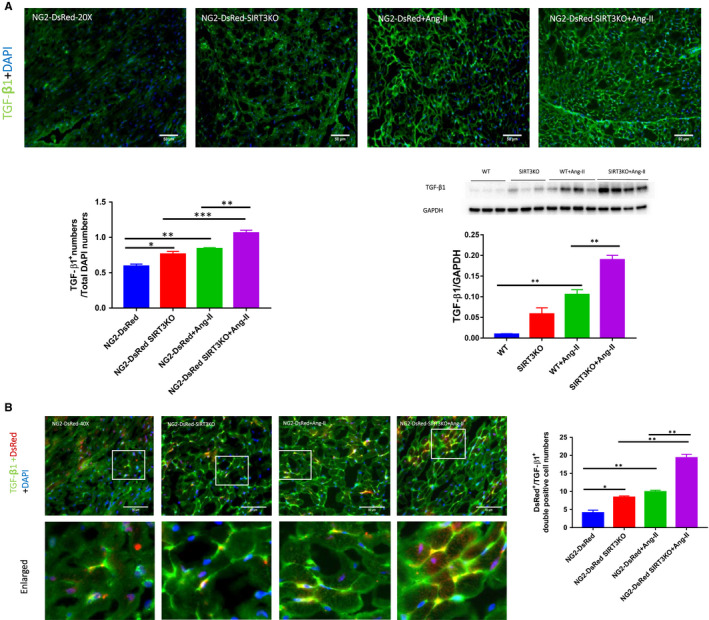
Knockout of SIRT3 further increased Ang‐II‐induced up‐regulation of TGF‐β1 expression. A, Knockout of SIRT3 sensitized Ang‐II‐induced up‐regulation of TGF‐β1. Immunostaining and Western blot analysis revealed a significant increase in TGF‐β1 expression in NG2‐DsRed mice or WT mice + Ang‐II (n = 4 mice). Knockout of SIRT3 further accentuated this up‐regulation. Mean ± SEM, *P < .05, **P < .01. B, Image revealing TGF‐β1^+^/DsRed^+^ pericytes presented in all groups of mice. The numbers of TGF‐β1^+^/DsRed^+^ double‐positive cell were increased in NG2‐DsRed mice + Ang‐II (n = 4) compared to NG2‐DsRed mice (n = 3 mice), and knockout of SIRT3 further increased numbers of TGF‐β1^+^/DsRed^+^ pericyte. Mean ± SEM, *P < .05, **P < .01

Immunostaining and Western blot analysis showed the levels of TGF‐β1 were significantly up‐regulated in the hearts of WT mice + Ang‐II compared to WT mice. Knockout of SIRT3 further enhanced expression of TGF‐β1 compared to WT mice + Ang‐II (Figure 5A).

Using pericyte tracing NG2‐DsRed mouse, we also found that TGF‐β1^+^/DsRed^+^ cells were presented in mouse hearts (Figure 5B). The numbers of TGF‐β1^+^/DsRed^+^ cell were increased in the NG2‐DsRed mice + Ang‐II than the NG2‐DsRed mice. Knockout of SIRT3 caused further increase in TGF‐β1^+^/DsRed^+^ cells (Figure 5B). There were staining areas of TGF‐β1^+^/DsRed^+^ around mouse coronary arteries. Both Ang‐II infusion and SIRT3KO resulted in increased areas of TGF‐β1^+^/DsRed^+^ compared to NG2‐DsRed mice (Figure S2C).

### 3.8 Knockout of SIRT3 augmented Ang‐II‐induced ROS formation

**FIGURE 6:**
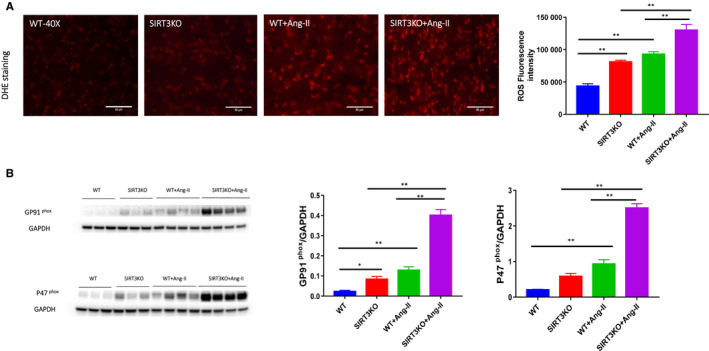
Knockout of SIRT3 promoted Ang‐II‐induced NADPH oxidase‐derived ROS formation. A, With immunostaining of DHE, there was a significant increase of integrated density in WT mice + Ang‐II (n = 4 mice) compared to WT mice (n = 3 mice). Integrated density was further increased in SIRT3KO mice + Ang‐II (n = 4) in comparison with WT mice + Ang‐II (n = 4 mice). Mean ± SEM, *P < .05, **P < .01. B, Western blot analysis revealed that Ang‐II up‐regulated expression of gp91^phox^ and p47^phox^ in the hearts. These gene expressions were significantly up‐regulated by knockout of SIRT3 in the Ang‐II‐treated hearts. Mean ± SEM, *P < .05, **P < .01

The levels of ROS formation measured by DHE staining were significantly higher in the WT mice + Ang‐II than that of the WT mice. Ang‐II‐induced ROS formation was further enhanced in SIRT3KO mice + Ang‐II (Figure 6A). In addition, the expression of gp91^phox^ and p47^phox^ was up‐regulated in the WT mice + Ang‐II compared to the WT mice. Knockout of SIRT3 further enhanced the expression of gp91^phox^ and p47^phox^ in comparison with WT mice + Ang‐II (Figure 6B).

## 4 DISCUSSION

In the present study, we found that Ang‐II infusion led to reduction of SIRT3 expression in mouse hearts. Meanwhile, knockout of SIRT3 exacerbated Ang‐II‐induced cardiac fibrosis and hypertrophy. This was accompanied by a significant reduction of CFR and cardiac dysfunction. Furthermore, Ang‐II‐induced pericyte‐myofibroblast/fibroblast transition was further enhanced by knockout of SIRT3, which contributed to the cardiac fibrosis. Ang‐II infusion also resulted in increased NADPH oxidase expression and ROS formation, which were further increased by knockout of SIRT3. In addition, Ang‐II‐induced expression of TGF‐β1 in pericytes was enhanced in SIRT3 KO mice, suggesting this may be associated with the pericyte‐myofibroblast/fibroblast transition.^21^, ^26^, ^27^ Overall, our data demonstrated that Ang‐II led to increased cardiac fibrosis partly via regulating SIRT3‐mediated pericyte‐myofibroblast/fibroblast transition and ROS‐TGF‐β1 pathway.

In the present study, we confirmed the effect of Ang‐II on cardiac remodelling including cardiac fibrosis, increased coronary artery thickness and cardiac hypertrophy. Cardiac dysfunction was followed. Accumulating evidence suggests that cardiac fibrosis results in cardiomyocyte hypertrophy and cardiac dysfunction.^6^, ^28^ Hypertension‐mediated coronary remodelling, which may lead to the reduced CFR, makes the heart more vulnerable to cardiomyocyte death and eventually heart failure.^5^ These two structural alterations interact with each other and contribute to the development of cardiac dysfunction and heart failure.^29^ Our study showed that Ang‐II infusion significantly reduced SIRT3 levels in the mouse hearts. Furthermore, Ang‐II‐induced myocardial fibrosis, capillary rarefaction and cardiomyocyte hypertrophy were accentuated by knockout of SIRT3. This was accompanied by increased IVRT and decreased levels of EF and FS. Although Ang‐II infusion alone did not cause a significant reduction of CFR, it caused a significant reduction of CFR in SIRT3KO mice. Taken together, our data suggested that a reduction of SIRT3 may contribute to Ang‐II‐mediated cardiac remodelling and heart failure.

Pericytes are support cells around capillaries, pre‐capillary arterioles, post‐capillary venules and collecting venules.^30^ Increasing evidence suggests that pericytes contribute to the underlying pathogenesis of fibrosis.^31^, ^32^ In NG2 tracing reporter NG2‐DsRed mouse, NG2^+^ pericyte express DsRed (red colour) provided a more reliable approach for tracing pericyte.^33^, ^34^, ^35^ In the present study, the numbers of NG2‐DsRed cells were increased significantly by Ang‐II infusion, while knockout of SIRT3 further increased pericytes in the mouse hearts. Our data further confirmed the contribution of SIRT3 to Ang‐II‐mediated pericyte recruitment by staining with DsRed and PDGFR‐β. The key question of our present study was to determine whether these increased pericytes contributing to cardiac fibrosis. Several lines of evidence show that pericyte‐to‐myofibroblast/fibroblast transition is one of the main sources of fibrosis in kidney and tumours,^15^, ^20^, ^21^ while few studies examined the role of pericytes in hypertensive heart.^20^, ^21^, ^26^, ^30^ Typically, pericyte‐myofibroblast/fibroblast transition was examined through double staining of both pericyte and fibrosis markers such as FSP‐1 and α‐SMA.^20^, ^21^, ^26^, ^30^ FSP‐1 is a highly specific marker for fibroblast, while α‐SMA expression is a key feature of myofibroblast marker.^36^, ^37^ To tracing NG2‐DsRed pericyte‐myofibroblast/fibroblast transition, we double‐stained DsRed (pericyte original) with FSP‐1 and α‐SMA. The FSP‐1^+^/DsRed^+^ or α‐SMA^+^/DsRed^+^ double‐positive cells were presented in the mouse hearts, indicating the process of pericyte‐myofibroblast/fibroblast transition occurred. Previous studies showed that FSP‐1 and α‐SMA could be also expressed in endothelial cells and arteriolar pericytes, respectively.^38^, ^39^ In our study, no IB4^+^/FSP‐1^+^ double‐positive cells were found in the mouse heart (Figure S4), indicating that FSP‐1 was not derived from endothelial cells or ECs do not transform into fibrosis. Moreover, most α‐SMA^+^ cells were presented in interstitial tissues, but not found around arterioles (Figures 3B and 4B), suggesting these α‐SMA^+^ cells were myofibroblast. Importantly, the numbers of FSP‐1^+^/DsRed^+^, collagen‐I^+^/NG2^+^ and α‐SMA^+^/DsRed^+^ cells were increased by Ang‐II infusion and further enhanced by knockout of SIRT3. These indicated that knockout of SIRT3 further promoted Ang‐II‐mediated pericyte‐myofibroblast/fibroblast transition and was coherent with increased cardiac fibrosis. Although our present data suggested a potential role of pericyte transition in the hearts, some controversies still exist regarding if pericytes as mesenchymal stem cells. Previous study had challenged the notion of pericyte as resident progenitors in multitudes of tissues.^40^ Notably, this study was done with PDGFRβ‐Cre mouse, which was found not being very suitable for tracing pericyte.^40^ Here, we used NG2‐DsRed reporter mouse to trace NG2^+^ pericyte, which has been proved to be practicable by many studies.^34^, ^35^, ^41^, ^42^ Taken together, our study provided a direct evidence that SIRT3 had a regulatory role in the Ang‐II‐induced pericyte‐myofibroblast/fibroblast transition in the mouse hearts.

Pericytes are vascular mural cells of mesenchymal origin, embedded in the basement membrane of microvasculature, where they make specific local contacts with endothelium.^13^, ^14^, ^16^, ^17^, ^18^ Pericytes are a subpopulation of mesenchymal stem cells (MSCs), which can differentiate into classic MSC triads, that is osteoblasts and vascular smooth muscle cells (VSMCs) and fibroblasts. In the NG2 tracing reporter mouse, we found that NG2‐DsRed pericytes also existed within coronary artery wall. This is consistent with previous study showing pericytes worked as progenitor of smooth muscle and contributed coronary artery formation.^42^ Furthermore, we found that infusion with Ang‐II resulted in an increase in NG2‐DsRed pericytes in coronary artery together with increased thickness. Ang‐II‐induced increase in wall thickness was further developed by knockout of SIRT3. In addition, Ang‐II‐induced perivascular fibrosis of coronary arteries was increased in SIRT3KO mice. By double staining of FSP‐1 and DsRed, we found that the area of FSP‐1^+^/DsRed^+^ around coronary arteries was increased by Ang‐II infusion as well as knockout of SIRT3. Moreover, Ang‐II‐induced decreased capillary density was accentuated by knockout of SIRT3, contributing to the coronary artery remodelling and microvascular rarefaction. These data suggested a potential role of Ang‐II and SIRT3 in the development of perivascular fibrosis via pericyte‐myofibroblast/fibroblast transition. Additionally, knockout of SIRT3 exacerbated Ang‐II‐induced coronary remodelling and caused impairment of CFR. These structural changes might reduce the compliance of coronaries and increase its resistance. Consequently, the blood perfusion from coronaries decreased leading to a decrease in CFR.

TGF‐β1 is the key mediator of cardiac fibrosis and contributes to fibroblast proliferation and extracellular matrix (ECM) accumulation.^6^, ^43^ Besides, TGF‐β1 is also an important regulator for the pericyte differentiation to myofibroblast and fibrosis.^21^, ^26^, ^27^ In our study, knockout of SIRT3 further increased Ang‐II‐induced TGF‐β1 expression, which is in line with the alterations of pericyte‐myofibroblast/fibroblast transition. Previous studies showed that TGF‐β1 was derived from endothelial cells, vascular smooth muscles and pericytes.^44^, ^45^ We found TGF‐β1^+^/DsRed^+^ double‐positive cells presented in the mouse hearts, suggesting that pericytes may be one of resources contributing to TGF‐β1 production. Interestingly, infusion of Ang‐II increased the numbers of TGF‐β1^+^/DsRed^+^ cell, and knockout of SIRT3 further enhanced this increase, indicating the role of SIRT3 and Ang‐II in pericytes producing TGF‐β1. Our data provided further evidence that Ang‐II and SIRT3 may be associated with the pericyte‐myofibroblast/fibroblast transition by TGF‐β1 signalling in the heart.

In addition, we found that knockout of SIRT3 enhanced Ang‐II‐induced ROS formation together with an up‐regulation of gp91^phox^ and p47^phox^ expression. Gp91^phox^ and p47^phox^ represent levels of NADPH oxidase‐derived ROS formation.^10^, ^46^ ROS‐TGF‐β1 axis is considered as the essential underlying mechanisms of fibrosis.^47^, ^48^, ^49^ We therefore speculated that increased ROS levels in SIRT3KO mice may be partly explaining the up‐regulation of TGF‐β1 in this study.

## 5 CONCLUSION

**FIGURE 7:**
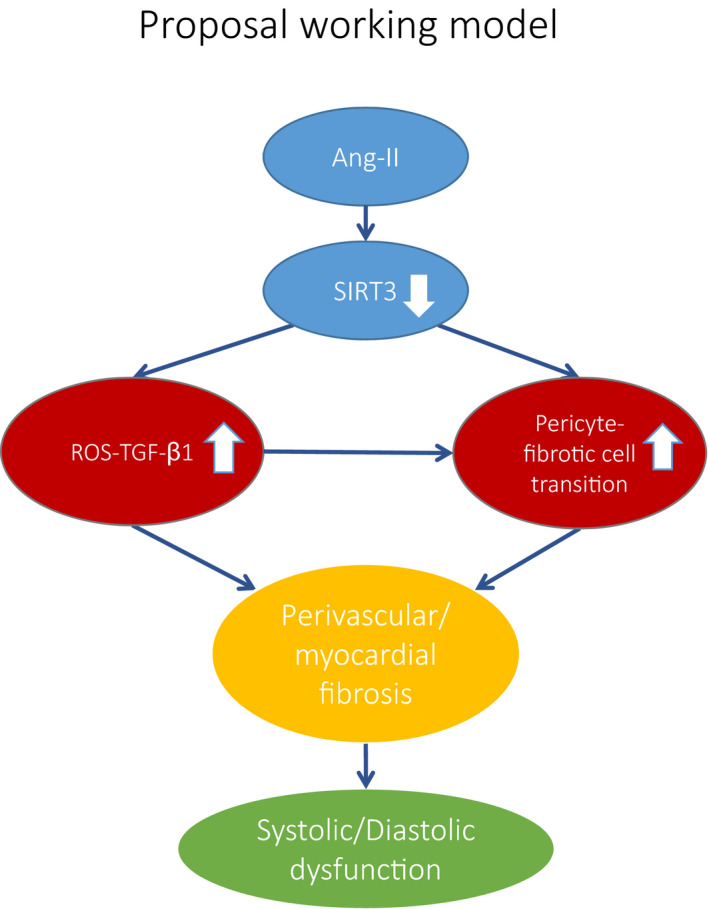
Proposal working model of Ang‐II‐induced fibrosis via suppressing SIRT3. Ang‐II causes a reduction of SIRT3 expression in the hearts. Reduction of SIRT3 leads to a significant increase in ROS formation and TGF‐β expression. Reduction of SIRT3 also promotes pericyte‐myofibroblast transitions. These abnormalities result in perivascular fibrosis and myocardial fibrosis, which caused a cardiac dysfunction

We concluded that SIRT3 has a critical role in Ang‐II‐induced cardiac fibrosis and remodelling through pericyte transition and ROS‐TGF‐β1 pathway (Figure 7).

### 5.1 Perspectives

In this study, we demonstrate that reduction of SIRT3 levels sensitized Ang‐II‐induced fibrosis through pericyte transition and ROS‐TGF‐β1 pathway. These changes not only result in increased fibrosis but also hypertrophy, which lead to cardiac remodelling and dysfunction eventually. Our study suggests a potential role of SIRT3 as a therapeutic target for cardiac fibrosis in hypertensive heart.

## CONFLICT OF INTEREST

The authors confirm that there are no conflicts of interest.

## AUTHOR CONTRIBUTION

**Han Su:** Data curation (lead); Formal analysis (lead); Investigation (lead); Methodology (equal); Writing‐original draft (equal). **heng zeng:** Data curation (equal); Formal analysis (equal); Investigation (equal); Methodology (equal); Project administration (lead); Supervision (equal); Writing‐original draft (equal). **Bo liu:** Data curation (supporting); Formal analysis (supporting). **Jian‐Xiong chen:** Conceptualization (lead); Funding acquisition (lead); Investigation (lead); Project administration (lead); Resources (lead); Supervision (lead); Writing‐review & editing (lead).

## Supporting information

Fig S1‐S4

## Data Availability

The data that support the findings of this study are available in the supplementary material of this article.
