# Correction to ‘Sirtuin 3 Is Essential for Hypertension‐Induced Cardiac Fibrosis via Mediating Pericyte Transition’

**DOI:** 10.1111/jcmm.71388

**Published:** 2026-10-03

Su H , Zeng H , Liu B , Chen J‐X . Sirtuin 3 Is Essential for Hypertension‐Induced Cardiac Fibrosis via Mediating Pericyte Transition. Journal of Cellular and Molecular Medicine 2020; 24: 8057–8068. 10.1111/jcmm.15437 PMC734816932463172

In Figure 6A of the DHE‐stained image for the control WT group, the image was incorrect. The correct image is shown below.

Figure 6A.

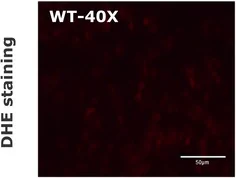

In Figure 5A, the same GAPDH band as in Figure 2D was used, as TGF‐β1 and β‐MHC were from the same PVDF membrane and shared the same GAPDH band.

Figure 5A and 5B (NG2‐DsRed mice). (A) Low‐magnification (20X) overview showing the regional expression profile of TGF‐β1 (green); the co‐stained cell marker channel is omitted here for visual clarity of overall expression levels. (B) High‐magnification (40X) individual channels and merged view of the identical tissue section, demonstrating co‐localization of TGF‐β1 (green) with NG2‐DsRed (red) to identify the cell source. The bounding box indicates the region magnified in Panel B.

We apologize for this error.

