# Supplementary material for: Sirtuin 3 is essential for hypertension‐induced cardiac fibrosis via mediating pericyte transition
Source: J Cell Mol Med. 2020 May 28;24(14):8057–68. doi: 10.1111/jcmm.15437 (PMC7348169; doi:10.1111/jcmm.15437)
Supplement: Supplementary file 1 — Fig S1‐S4 [file JCMM-24-8057-s001.docx]

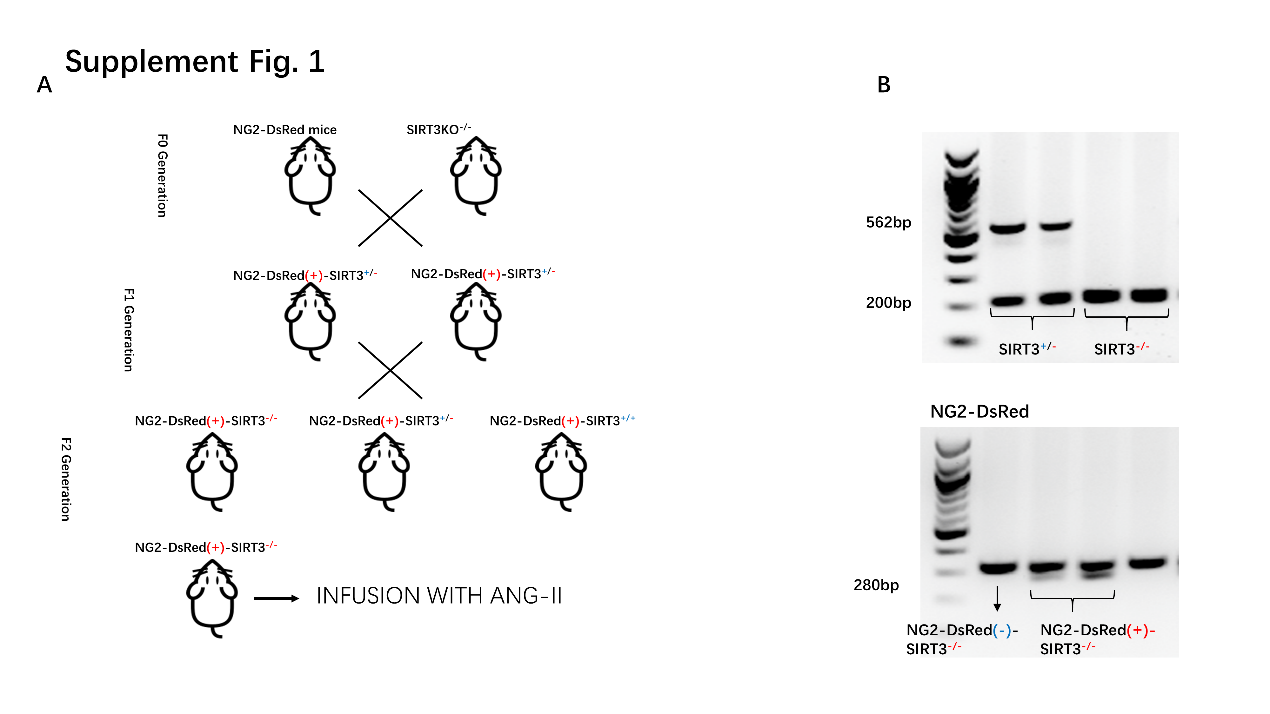


**Figure S1.** Generation of NG2-DsRed-SIRT3KO mice. **A)** Scheme of NG2-DsRed mice crossing with SIRT3KO mice. **B)** Through PCR, there was a band of 200bp in Homozygous SIRT3KO mice (n=5), heterozygous SIRT3KO mice showed bands of both 562bp and 200bp (n=6). There was one band of 280bp in NG2-DsRed-SIRT3KO mice, there was no band in 280bp in non-NG2-DsRed mice.


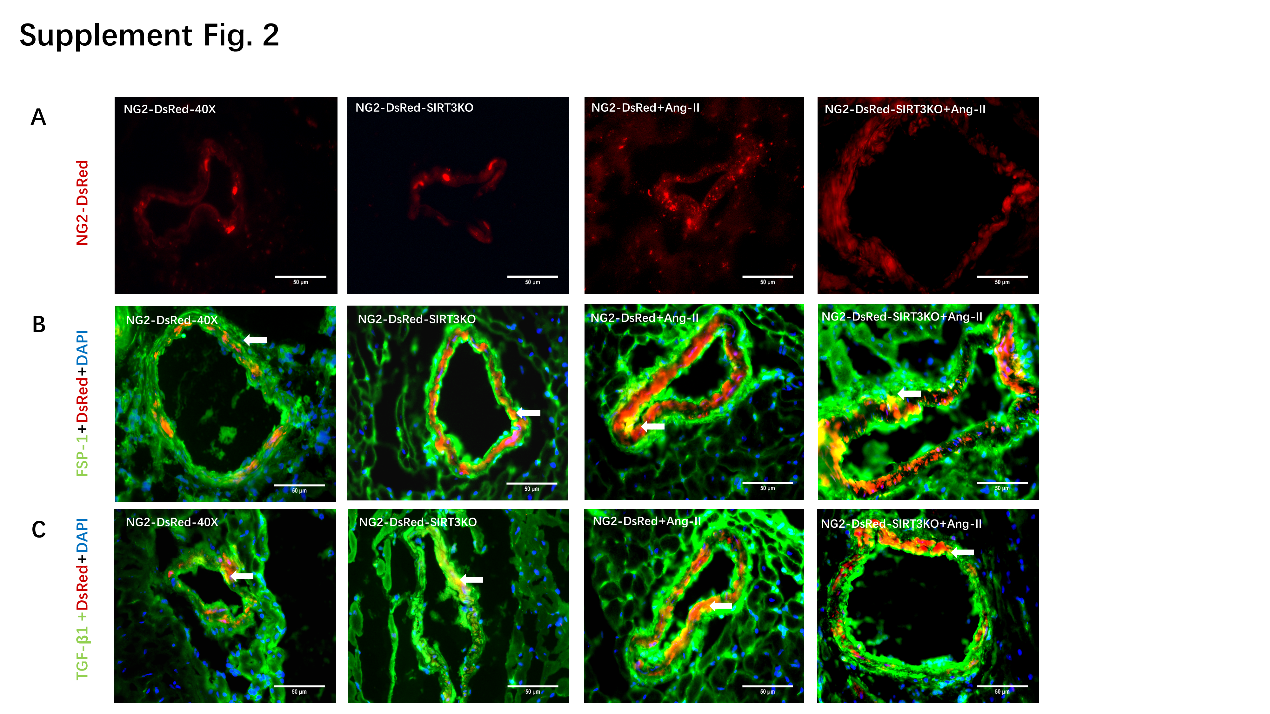


**Figure S2.** Knockout of SIRT3 further developed Ang-II-induced perivascular fibrosis via pericyte-fibroblast transition. **A)** We found NG2-DsRed^+^ residing around coronary arteries. **B)** Deletion of SIRT3 and Ang-II both increased FSP-1^+^/DsRed^+^ area around coronary arteries. **C)** Deletion of SIRT3 and Ang-II both increased TGF-β1^+^/DsRed^+^ area around coronary arteries.


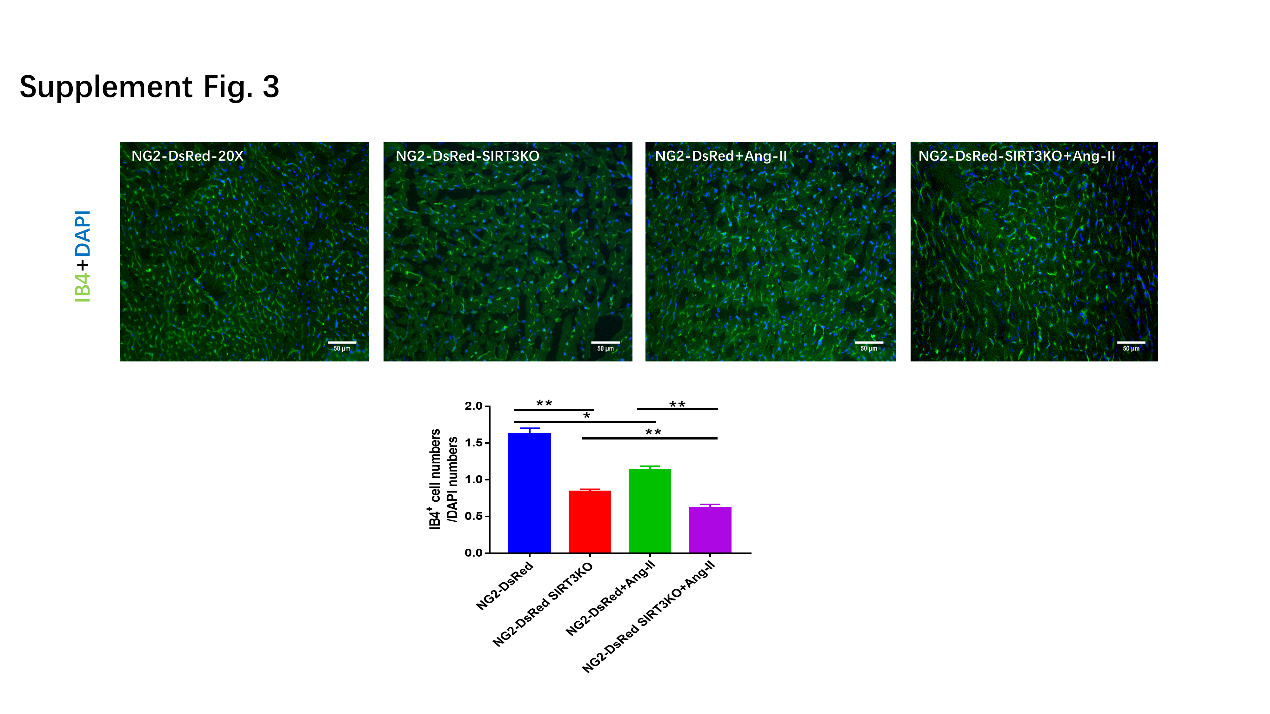


**Figure S3.** Immunostaining revealed that Ang-II-induced decrease of IB4 was further developed by knockout of SIRT3. Mean ± S.E.M., *P<0.05, **P<0.01.


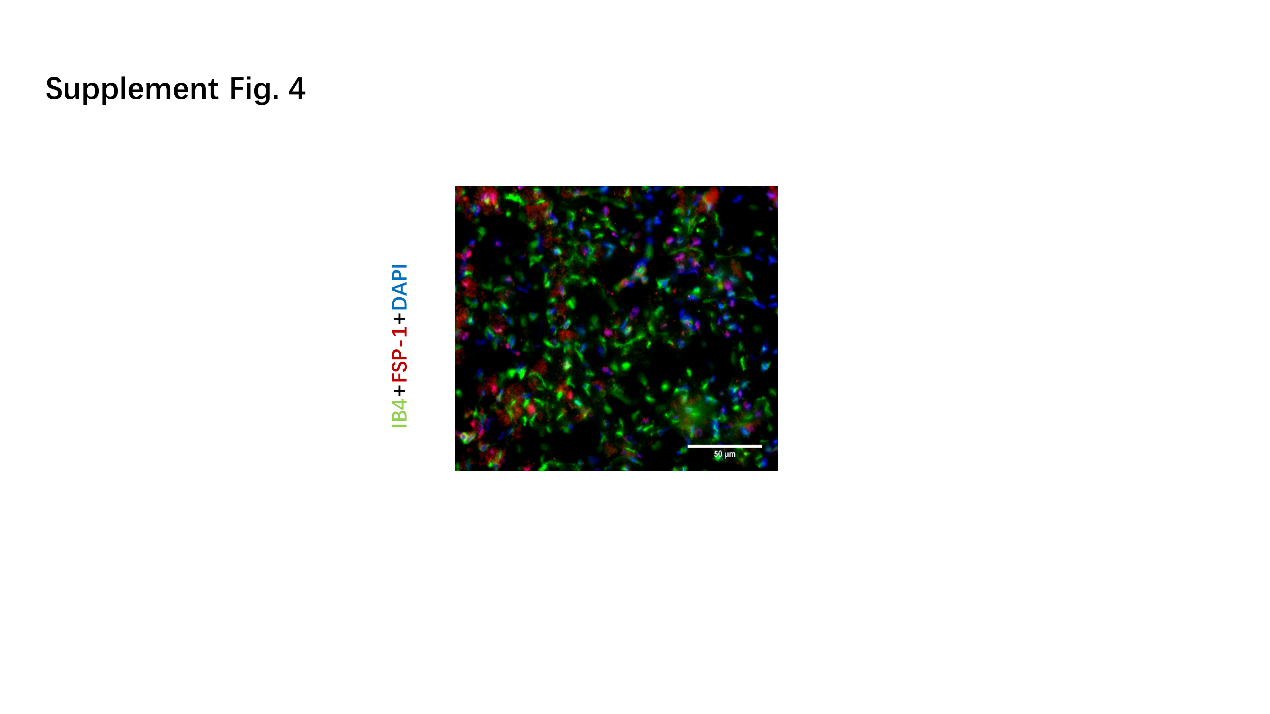


**Figure S4.** Immunostaining revealed that no IB4^+^/FSP-1^+^ was found in heart tissue.
